# *NTRK* gene aberrations in triple‐negative breast cancer: detection challenges using IHC, FISH, RT‐PCR, and NGS

**DOI:** 10.1002/cjp2.324

**Published:** 2023-05-04

**Authors:** Federica Zito Marino, Simona Buono, Marco Montella, Rosa Giannatiempo, Francesco Messina, Giovanni Casaretta, Grazia Arpino, Giulia Vita, Francesco Fiorentino, Luigi Insabato, Alessandro Sgambato, Michele Orditura, Renato Franco, Marina Accardo

**Affiliations:** ^1^ Pathology Unit, Department of Mental and Physical Health and Preventive Medicine University of Campania “L. Vanvitelli” Naples Italy; ^2^ Ospedale Evangelico Betania Naples Italy; ^3^ Department of Clinical Medicine and Surgery University of Naples Federico II Naples Italy; ^4^ Anatomical Pathology Department, IRCCS CROB Rionero in Vulture Italy; ^5^ Pathology Unit S.M. delle Grazie Hospital Naples Italy; ^6^ Department of Advanced Biomedical Sciences, Pathology Section University of Naples “Federico II” Naples Italy; ^7^ Scientific Direction, Centro di Riferimento Oncologico della Basilicata (IRCCS‐CROB) Rionero in Vulture Italy; ^8^ Division of Medical Oncology, Department of Precision Medicine, School of Medicine University of Campania “L. Vanvitelli” Naples Italy

**Keywords:** triple‐negative breast carcinoma, neurotrophic tyrosine receptor kinase (NTRK), *NTRK* fusions, *NTRK* amplification, IHC, FISH, RT‐PCR, NGS

## Abstract

Triple‐negative breast cancer (TNBC) is usually an aggressive disease with a poor prognosis and limited treatment options. The neurotrophic tyrosine receptor kinase (NTRK) gene fusions are cancer type‐agnostic emerging biomarkers approved by the Food and Drug Administration (FDA), USA, for the selection of patients for targeted therapy. The main aim of our study was to investigate the frequency of *NTRK* aberrations, i.e. fusions, gene copy number gain, and amplification, in a series of TNBC using different methods. A total of 83 TNBCs were analyzed using pan‐TRK immunohistochemistry (IHC), fluorescence *in situ* hybridization (FISH), real‐time polymerase chain reaction (RT‐PCR), and RNA‐based next‐generation sequencing (NGS). Of 83 cases, 16 showed pan‐TRK positivity although no cases had *NTRK*‐fusions. Indeed, FISH showed four cases carrying an atypical *NTRK1* pattern consisting of one fusion signal and one/more single green signals, but all cases were negative for fusion by NGS and RT‐PCR testing. In addition, FISH analysis showed six cases with *NTRK1* amplification, one case with *NTRK2* copy number gain, and five cases with *NTRK3* copy number gain, all negative for pan‐TRK IHC. Our data demonstrate that IHC has a high false‐positive rate for the detection of fusions and molecular testing is mandatory; there is no need to perform additional molecular tests in cases negativity for NTRK by IHC. In conclusion, the *NTRK* genes are not involved in fusions in TNBC, but both copy number gain and amplification are frequent events, suggesting a possible predictive role for other NTRK aberrations.

## Introduction

Breast cancer is a clinically and genetically heterogeneous disease. Approximately 10–20% of breast cancers are triple‐negative breast cancer (TNBC), a subtype characterized by immunohistochemical lack of estrogen receptor (ER), progesterone receptor (PgR), and human epidermal growth factor receptor 2 (HER2) overexpression [1, 2, 3]. TNBCs have aggressive clinical behavior and poor prognosis since they are not responsive to either hormonal therapy or HER2‐specific inhibitors. The definition of new biomarkers with potential therapeutic implications represents one of the greatest challenges in the treatment of this subgroup of breast cancers.

In recent years, the neurotrophic tyrosine receptor kinase (*NTRK*) gene fusions have become new predictive biomarkers in several cancer types since the specific inhibitors have been developed and approved [4, 5]. Physiologically, the *NTRK1*, *NTRK2*, and *NTRK3* genes encode for a family of receptor tyrosine kinases (Trk) that play a role in neuronal development, function, survival, and proliferation. *NTRK* gene fusions have been shown to be a driver mechanism in various tumors, although at a very low incidence [6]. *NTRK* fusions can be detected using different methods including immunohistochemistry (IHC), fluorescence *in situ* hybridization (FISH), real‐time‐polymerase chain reaction (RT‐PCR), and RNA‐based next‐generation sequencing (NGS) [6]. ESMO, the Japan Society of Clinical Oncology and the Japanese Society of Medical Oncology have proposed the recommendation of *NTRK* fusion identification, suggesting the use of IHC as a prescreening method in tumors with a low incidence with mandatory molecular confirmation of IHC positive cases [7, 8]. The prescreening test based on IHC has been suggested because of the high specificity and sensitivity of pan‐TRK IHC observed in several tumors [9]. On the other hand, several studies have shown discordant results between IHC and the other molecular methods of *NTRK* detection in various solid tumors [10, 11, 12, 13]. A previous study evaluated *NTRK* fusions in TNBC patient samples using IHC as a prescreening method and the other molecular methods to confirm the *NTRK* fusion [14]. NTRK IHC resulted in a high false‐positive rate of *NTRK* gene fusion suggesting that another molecular assay should be recommended for *NTRK* fusion detection.

Beyond *NTRK* fusions, Lee *et al* showed that the *NTRK* genes could be affected by amplification in various cancer types [10]. However, the possible predictive role of *NTRK* gene amplification is still unclear, and further studies are needed to confirm whether *NTRK*‐amplified tumors are suitable for treatment with specific inhibitors. The main aim of this study was to assess the frequency of *NTRK* gene aberrations, such as fusions, copy number gain, and amplification, in a TNBC series. The analysis was performed using different assays including IHC, FISH, RT‐PCR, and RNA‐based NGS.

## Materials and methods

### Case selection and tumor specimen collection

A series of 109 triple‐negative breast tumor tissue samples from surgical resections performed between 2019 and 2022 at the University of Campania ‘L. Vanvitelli’, the Ospedale Evangelico Betania, the University of Naples ‘Federico II’, the S.M. delle Grazie Hospital, and the IRCCS CROB were collected. We retrospectively recorded clinical and pathological parameters, including the age of the patient at initial diagnosis, the histological type, the grade, and the stage. We defined TNBC according to the current guidelines: negative for ER or PgR if <1% or 0% of tumor cell nuclei are immunoreactive and HER2 negativity defined as either IHC expression of 0–1+ or lack of gene amplification by FISH [15]. We used four cases of secretory breast cances harboring *NTRK3* rearrangement as positive controls.

### Immunohistochemistry

IHC was performed on 4 μm paraffin‐embedded whole tissue sections for each case. Pan‐TRK immunohistochemical staining for TrkA/B/C expression was performed using Ventana pan‐TRK antibody (clone EPR17341; #790‐7026, ready to use, Ventana Medical Systems, Tucson, AZ, USA), a rabbit recombinant monoclonal antibody reactive to a C‐terminal epitope conserved across TRK‐A, ‐B, and ‐C proteins and present in both wild type and chimeric proteins. All assays were performed on a fully automated BenchMark XT device (Ventana Medical Systems). Ganglia of the submucosal plexus of a normal vermiform appendix and an infantile fibrosarcoma with known *ETV6‐NTRK3* fusion were used as positive controls and were run simultaneously with each sample. Lymphocytes served as internal negative controls. Tumors were considered positive if ≥1% of tumor cells exhibited positivity at any intensity above background. Different subcellular staining patterns were considered positive, as previously suggested (cytoplasmic, membranous, nuclear, and perinuclear) [8, 13, 14]. Signal intensity was expressed as a score, from 1 to 3, corresponding to weak, moderate, and strong signals. Two independent observers carried out immunohistochemical analysis, and both observers were blinded; in discordant cases, a consensus was reached by collegial discussion.

### Fluorescence *in situ* hybridization

FISH was carried out on three 4‐μm‐thick sections cut from each formalin‐fixed paraffin‐embedded (FFPE) sample using the BOND FISH kit (Leica Biosystems, Newcastle Upon Tyne, UK) on the automated BOND system (Leica Biosystems) according to the manufacturer's instructions. This kit consists of a formamide mixture to reduce nonspecific hybridization of nucleic acid probes. *NTRK1*, *NTRK2*, and *NTRK3* fusion detection was performed by three separate assays using specific break‐apart probes for each gene: ZytoLight SPEC NTRK1 Dual Color Break Apart Probe (ZytoVision, Bremerhaven, Germany); ZytoLight SPEC NTRK2 Dual Color Break Apart Probe (ZytoVision, Bremerhaven, Germany); ZytoLight SPEC NTRK3 Dual Color Break Apart Probe (ZytoVision, Bremerhaven, Germany). Slides were counterstained with 4′,6‐diamidino‐2‐phenylindole dihydrochloride (DAPI) in antifade solution and examined using an automated CytoVision platform (Leica Biosystems). FISH interpretation was performed with the automated fluorescence microscope Leica DM5500 B (Leica Biosystems) using the filter ET‐D/O/G for double Spectrum Green plus Spectrum Orange. FISH signals were counted in at least 100 nonoverlapping intact nuclei.

### *NTRK* fusion interpretation

FISH was considered positive in relation to two different patterns: (1) a classic break‐apart pattern with one fusion signal and two separated 3′ orange and 5′ green signals (separation distance of at least two signal diameters between the green and orange signals); (2) an atypical pattern with one fusion signal and a single orange signal without a corresponding green signal. Tumors were considered positive if ≥15% of tumor cells exhibited gene rearrangements.

### Interpretation of *NTRK* gene copy number gain and amplification

The mean copy numbers for *NTRK1*, *NTRK2*, and *NTRK3* genes were evaluated. In order to exclude polyploidy, FISH for centromeric alpha‐satellite sequences specific for chromosomes 1, 9, and 15, where the *NTRK1*, *NTRK2*, and *NTRK3* genes are located respectively, was performed on the cases showing copy number aberration of the genes. The FISH assay to evaluate polyploidy was performed using specific probes: Creative Bioarray CEN 1p FISH Probe Red; CytoCell Chromosome 9 Satellite III FISH Probe Aqua | OGT; CytoCell Chromosome 15 Alpha Satellite FISH Probe Red | OGT. In a normal interphase nucleus, two signals associated with the disomic status of chromosome 2 were expected; an increase in signals per nucleus indicated the polyploidy of the chromosome. The ratio between each *NTRK* gene mean copy number and the related CEP mean copy number was evaluated. Amplification of the *NTRK* genes was considered to occur when the *NTRK* locus‐specific probe/CEP ratio was ≥2; cases carrying *NTRK* copy number gain showed a *NTRK* locus‐specific probe/CEP ratio of <2 [15]. The criteria for copy number aberrations of *NTRK* genes were as follows: *NTRK* copy number gain with a mean copy number of 3–5 signals in ≥10% of cells and *NTRK* amplification with the presence of ≥6 copies of gene per cell in ≥10% of analyzed cells [16].

### RNA extraction

The hematoxylin and eosin‐stained tumor slides of each case were reviewed by a pathologist to select representative areas of tumor suitable for molecular testing. RNA extraction from FFPE tumor samples was carried out according to the manufacturers' protocols utilizing the MagCore Total RNA FFPE One‐Step Kit (RBC Bioscience Corp., New Taipei City, Taiwan) on the automated extraction system MagCore Super (RBC Bioscience Corp.) based on magnetic bead extraction technology.

### Real‐time PCR

The RNA isolated from FFPE tumor samples was used to analyze *NTRK1*, *NTRK2*, and *NTRK3* fusions. Real‐time‐PCR (RT‐PCR) was performed using the EasyPGX ready NTRK fusion kit (Diatech Pharmacogenetics, Jesi, Italy), on the EasyPGX qPCR instrument 96 (Diatech Pharmacogenetics). The detectable, but not distinguishable, gene fusions detected with this test are listed in supplementary material, Table S1. A positive and a negative control were used. The detection was based on the use of the fluorescent probes marked as follow: probes marked with FAM for the targets and probes marked with HEX for the endogenous control genes. The data were analyzed by EasyPGX Analysis Software (Diatech Pharmacogenetics).

### Next‐generation sequencing

RNA input quantification was measured by RT‐PCR on the EasyPGX qPCR instrument 96 (Diatech Pharmacogenetics), detecting two highly conserved RNA regions of 105 and 175 bp through two probes labeled with FAM and HEX, respectively. The RNA concentration was assessed by quantification with a standard curve in the HEX channel. The ratio between the quantification (ng/μl) obtained in FAM and that obtained in the HEX allows evaluation of the DNA fragmentation. The data were analyzed by EasyPGX Analysis Software (Diatech Pharmacogenetics) in order to evaluate the concentration and degree of fragmentation of the samples. The RNA libraries were generated using the Myriapod NGS Cancer Panel RNA (Diatech Pharmacogenetics), according to the manufacturer's instructions. The kit allows the detection of the main gene fusions involving 10 recurrently rearranged cancer genes: *ALK*, *ROS1*, *RET*, *NTRK1*, *NTRK2*, *NTRK3*, *FGFR2*, *FGFR3*, *PPARG*, and the skipping of exon 14 of *MET* in total RNA isolated from tumor tissue. The RNA was retro‐transcribed into cDNA using random hexamers. Subsequently, cDNA was amplified by multiplex PCR using two primer mixtures to obtain fragments between 47 and 184 bases, including fusions of interest and endogenous control genes (PCR1). The amplification products were purified with magnetic beads to remove residual primers. An amplification‐based indexing reaction (PCR2) followed, which allowed a unique pair of two sample‐specific barcodes (indexes) and an Illumina platform‐specific adapter to be attached to each fragment. The libraries thus constituted were normalized in quantity by magnetic beads to guarantee a homogeneous coverage of the samples during sequencing. Finally, the normalized libraries were mixed (library pool) and sequenced in parallel on the Illumina MiSeq platform (Illumina Inc., San Diego, CA, USA) with MiSeq Reagent Kit v2 Micro (300 cycles) flow cell (Illumina Inc.). The data generated by the sequencer were analyzed locally with dedicated Myriapod NGS Data Analysis Software (v 4.0.2; Diatech Pharmacogenetics).

### Statistical analysis

A Pearson chi‐square test was conducted using SPSS 20.0 for Mac (SPSS Inc., Chicago, IL, USA) to determine the association of *NTRK* aberrations with the clinical and pathological features.

## Results

### Clinical and pathological characteristics of patients

Overall, 109 TNBC patients were analyzed. The mean age of patients was 50 years (range 30–58 years); 84 (77%) were younger and 25 (23%) were older than 50 years of age. The cohort included 107 (98.2%) no special type carcinomas, 1 (0.9%) metaplastic carcinoma, and 1 (0.9%) pleomorphic lobular carcinoma; all cases were basal‐like. Of the 109 TNBC, 88 were grade (G)3 (80.7%) and 21 were grade G2 (19.3%). The postoperative pathological stages were I in 45 cases (41.2%), II in 45 cases (41.2%), and III in 19 cases (17.5%). The clinical and pathological features are summarized in Table 1.

**Table 1:** *NTRK* aberrations and clinical–pathological features of the series analyzed

			*NTRK1* FISH			*NTRK*2 FISH	*NTRK3* FISH	*NTRK*‐fusion RNA‐based NGS		
	No. cases (%)	Pan‐TRK IHC positive	Isolated 5′	CNG	Amp	CNG	CNG	Positive	Negative	NP
Characteristics	109	25	4	1	6	1	5	0	84	25
Age										
≥50	25 (23%)	11 (44%)	2 (50%)	1 (100%)	2 (33.3%)	0	1 (20%)	0	18 (21.4%)	7 (28%)
<50	84 (77%)	14 (56%)	2 (50%)	0	4 (66.7%)	1 (100%)	4 (80%)	0	66 (78.6%)	18 (72%)
Histotype										
NST	107 (98.2%)	25 (100%)	4 (100%)	1 (100%)	6 (100%)	1 (100%)	5 (100%)	0	84 (100%)	25 (100%)
Metaplastic	1 (0.9%)	0	0	0	0	0		0		
Pleomorphic lobular	1 (0.9%)	0	0	0	0	0		0		
Grading										
G2	21 (19.3%)	5 (20%)	0	1 (100%)	1 (16.7%)	0	2 (40%)	0	11 (13.1%)	10 (40%)
G3	88 (80.7%)	20 (80%)	4 (100%)	0	5 (83.3%)	1 (100%)	3 (60%)	0	73 (86.9%)	15 (60%)
TNM stage										
pT1, pN0	45 (41.2%)	19 (76%)	3 (75%)	1 (100%)	4 (66.7%)	0	3 (60%)	0	40 (47.6%)	5 (20%)
pT2–pT3, pN0/pT1, pN1	45 (41.2%)	6 (24%)	0	0	2 (33.3%)	1 (100%)	1 (20%)	0	32 (38.1%)	13 (52%)
pT3, pN1/any T, pN2	19 (17.6%)	0	1 (25%)	0	0	0	1 (20%)	0	12 (14.3%)	7 (28%)
Neoadjuvant therapy										
No	66 (61%)	21 (84%)	4 (100%)	1 (100%)	5 (83.3%)	1 (100%)	4 (80%)	0	58 (69%)	8 (32%)
Yes	43 (39%)	4 (16%)	0	0	1 (16.7%)	0	1 (20%)	0	26 (31%)	17 (68%)
RCB										
0	0	0	–	–	0	–	–	0	0	0
I	4 (9.3%)	0	–	–	0	–	–	0	3 (11.5%)	1 (5.9%)
II	31 (72.1%)	3 (75%)	–	–	1 (100%)	–	1 (100%)	0	18 (69.3%)	13 (76.5%)
III	8 (18.6%)	1 (25%)	–	–	0	–	–	0	5 (19.2%)	3 (17.6%)

Amp, gene amplification; CNG, copy number gain; NP, not performed; NST, no special type; RCB, residual cancer burden.

### Pan‐TRK IHC

**Figure 1:**
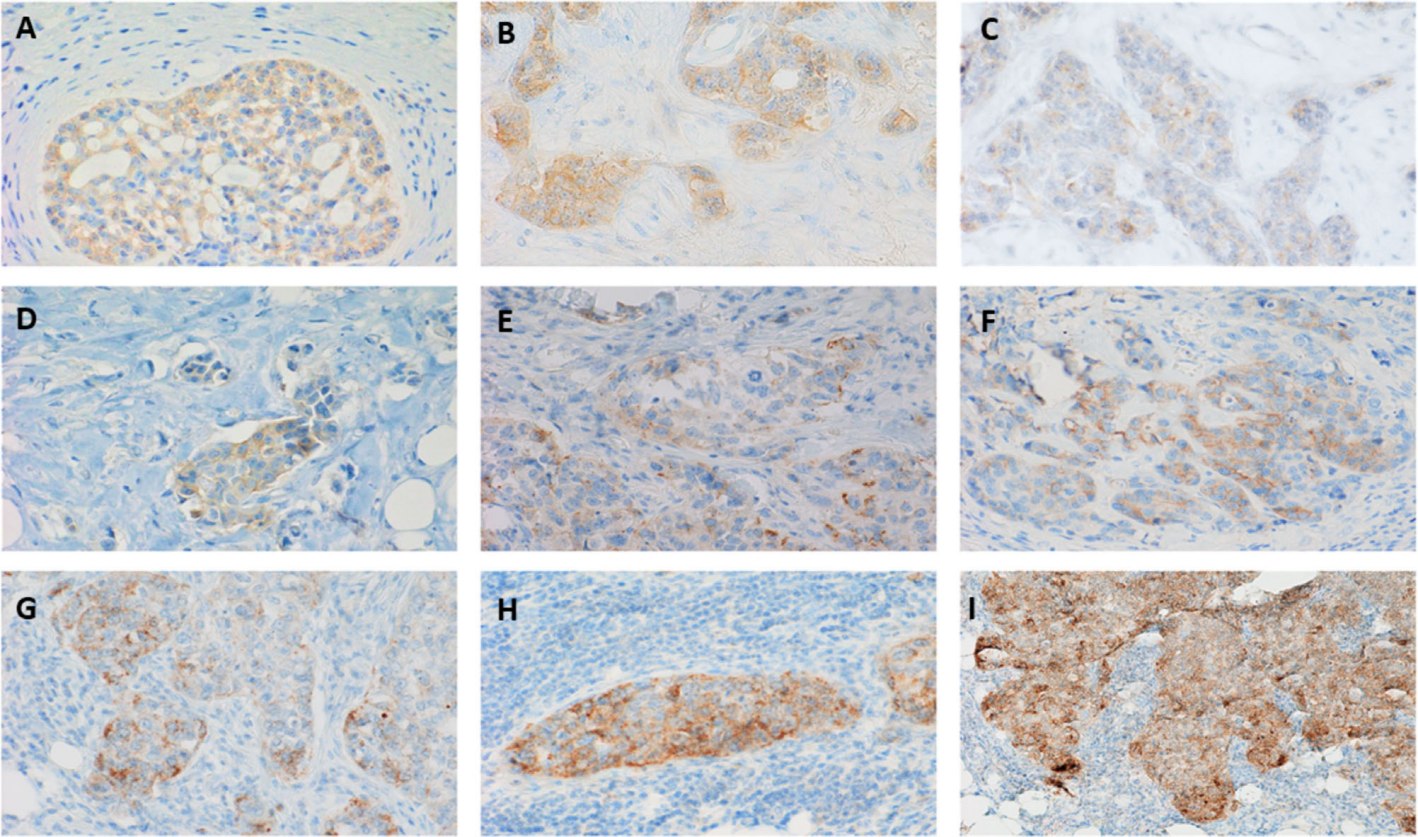
Representative pan‐TRK IHC results: (A–D) weak cytoplasmic immunoreactivity in neoplastic cells (original magnification ×40); (E–G) moderate cytoplasmic immunoreactivity in neoplastic cells (original magnification ×40); (H and I) strong cytoplasmic immunoreactivity in neoplastic cells (original magnification ×40).

Overall, 25 of 109 cases showed positive pan‐TRK immunoreactivity; the remaining 84 cases were negative. In all IHC‐positive samples, a signal was present in more than 20% of tumor cells and the staining was observed exclusively in the cytoplasm. Signal intensity was strong in 2 cases (score 3), moderate in 9 cases (score 2), and weak in 14 cases (score 1) (Figure 1). NTRK IHC positivity was not statistically associated with clinical–pathological features. All 4 cases of secretory breast cancer selected as controls were pan‐TRK positive (supplementary material, Figure S1).

### Fluorescence *in situ* hybridization

**Figure 2:**
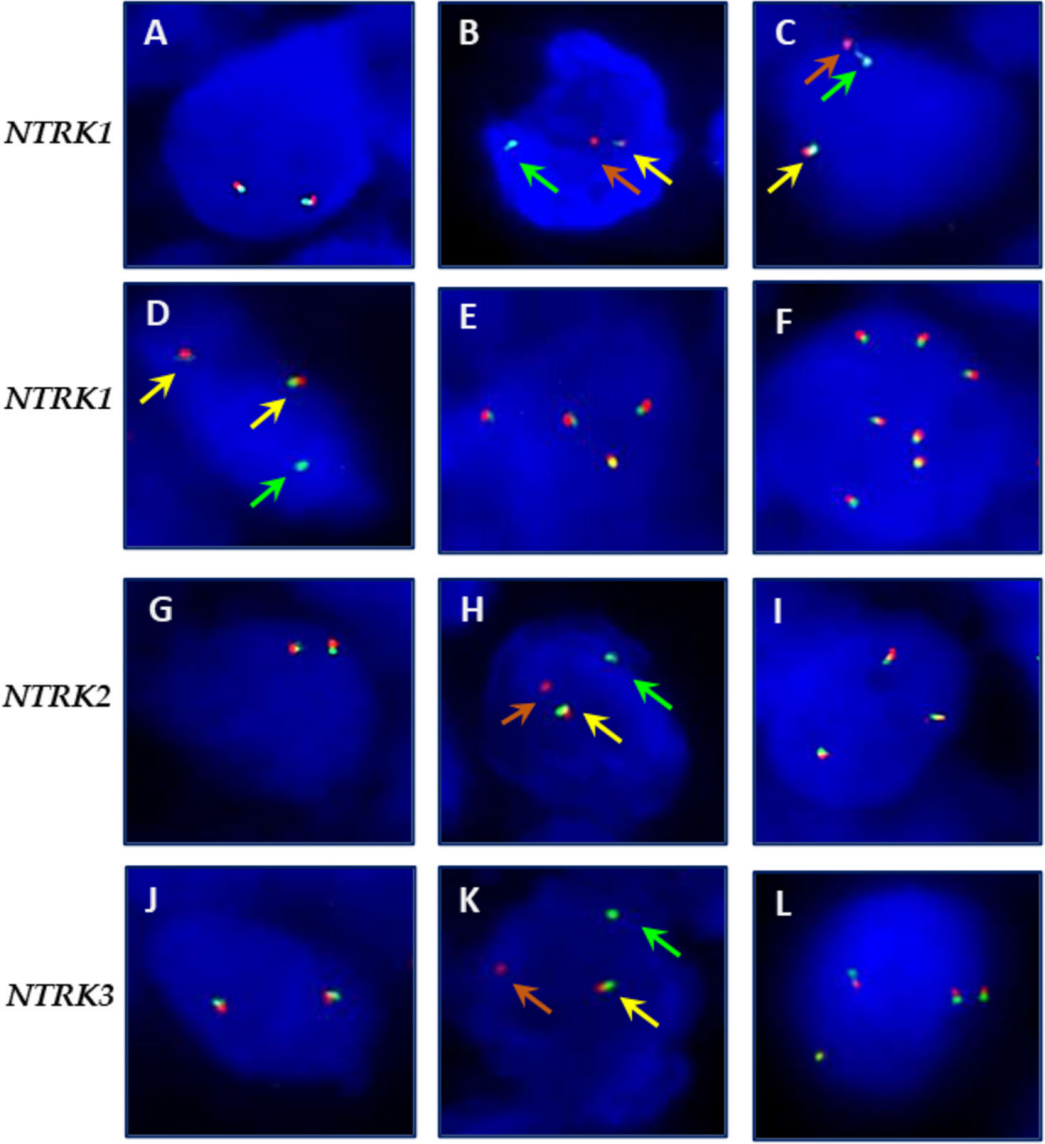
Representative *NTRK1*, *NTRK2*, and *NTRK3* FISH results: (A) *NTRK1* FISH showing the typical pattern wild type: two fusion signals (original magnification ×100); (B) *NTRK1* FISH showing the typical pattern of rearrangement: one fusion signal (yellow arrow) and split 3′ (orange arrow) and 5′ (green arrow) signals, with a separation distance of at least two signal diameters between the green (green arrow) and orange (orange arrow) signals (original magnification ×100); (C) *NTRK1* FISH showing one fusion signal (yellow arrow) and split 3′ (orange arrow) and 5′ (green arrow) signals, with a separation distance of less than two signal diameters between the green (green arrow) and orange signals (orange arrow) (original magnification ×100); (D) *NTRK1* FISH showing an atypical pattern: two fusion signals (yellow arrows) and an isolated 5′ signal (green arrow) (original magnification ×100); (E) *NTRK1* FISH showing copy number gain with four fusion signals (original magnification ×100); (F) *NTRK1* FISH showing gene amplification with seven fusion signals (original magnification ×100); (G) *NTRK2* FISH showing the typical wild type pattern: two fusion signals (original magnification ×100); (H) *NTRK2* FISH showing the typical pattern of rearrangement: one fusion signal (yellow arrow) and split 3′ (orange arrow) and 5′ (green arrow) signals, with a separation distance of at least two signal diameters between the green (green arrow) and orange signals (orange arrow) (original magnification ×100); (I) *NTRK2* FISH showing gene copy number gain with three fusion signals(original magnification ×100); (J) *NTRK3* FISH showing the typical wild type pattern: two fusion signals (original magnification ×100); (K) *NTRK3* FISH showing the typical pattern of rearrangement: one fusion signal (yellow arrow) and split 3′ (orange arrow) and 5′ (green arrow) signals, with a separation distance of at least two signal diameters between the green (green arrow) and orange signals (orange arrow)(original magnification ×100); (L) *NTRK3* FISH showing copy number gain with four fusion signals (original magnification ×100).

FISH tests were performed on all cases to evaluate *NTRK1*, *NTRK2*, and *NTRK3* fusions. In our series, no case was positive for *NTRK* fusions. Three cases showed *NTRK1* rearrangement in approximately 7% of tumor cells analyzed, but this percentage was not sufficient to indicate positivity. Ten cases showed *NTRK1* split‐apart signals that were less than two signal diameters apart in approximately 5–15% of tumor cells analyzed (Figure 2C). *NTRK1* FISH showed 4 of 109 cases carrying an atypical pattern, particularly 2 cases with one fusion signal and a single green signal and 2 cases carrying an atypical pattern with three‐four fusion signals and a single green signal (Figure 2D). Moreover, six cases showed *NTRK1* gene amplification and one case showed copy number gain; all cases showed disomy of chromosome 1 (Figure 2E,F). No atypical *NTRK2* signal patterns were detected in our series; one case showed copy number gain, with disomy of chromosome 9 (Figure 2I). No atypical *NTRK3* signal patterns were detected in our series; 5 of 109 cases showed copy number gain, with disomy of chromosome 15 (Figure 2L). *NTRK* FISH aberrations were not statistically associated with either clinical–pathological features or pan‐TRK expression. All 4 cases of secretory breast cancer selected as controls harbored rearranged *NTRK3* (supplementary material, Figure S1).

### Real‐time RT‐PCR

All cases were analyzed by RT‐PCR analysis. No *NTRK* fusions were detected.

### RNA‐based NGS

The NGS analysis was performed on 84 of 109 cases; unfortunately, 25 cases were not tested with NGS as the RNA quantity and the quality were not adequate for this assay. No *NTRK* fusions were identified in the cases analyzed. Among 25 cases that were pan‐TRK IHC positive, 23 were *NTRK1‐3* wild type and 2 cases were not analyzed by NGS (Table 2). Among four cases with an atypical *NTRK1* FISH pattern, three cases were *NTRK1* wild type and one case was not analyzed by NGS (Table 2).

**Table 2:** Comparison of different assays in cases carrying NTRK aberrations

		*NTRK* FISH				
Case	Pan‐TRK IHC score	*NTRK1*	*NTRK2*	*NTRK3*	RT‐PCR	RNA‐NGS
1	3	ap (4F 1G)	NR	NR	NR	wt
2	3	CNG	CNG	CNG	NR	wt
3	2	ap (1F 1G)	NR	CNG	NR	NP
4	2	NR	NR	CNG	NR	wt
5	2	NR	NR	NR	NR	wt
6	2	NR	NR	NR	NR	wt
7	2	NR	NR	NR	NR	wt
8	2	NR	NR	NR	NR	wt
9	2	NR	NR	NR	NR	wt
10	2	NR	NR	NR	NR	wt
11	2	NR	NR	NR	NR	wt
12	1	NR	NR	NR	NR	wt
13	1	NR	NR	NR	NR	wt
14	1	NR	NR	NR	NR	NP
15	1	NR	NR	NR	NR	wt
16	1	NR	NR	NR	NR	wt
17	1	NR	NR	NR	NR	wt
18	1	NR	NR	NR	NR	wt
19	1	NR	NR	NR	NR	wt
20	1	NR	NR	NR	NR	wt
21	1	NR	NR	NR	NR	wt
22	1	NR	NR	NR	NR	wt
23	1	NR	NR	NR	NR	wt
24	1	NR	NR	NR	NR	wt
25	1	NR	NR	NR	NR	wt
26	0	ap (1F 1G)	NR	NR	NR	wt
27	0	ap (3F 1G)	NR	NR	NR	wt
28	0	Amp	NR	CNG	NR	NP
29	0	Amp	NR	CNG	NR	NP
30	0	Amp	NR	NR	NR	NP
31	0	Amp	NR	NR	NR	NP
32	0	Amp	NR	NR	NR	NP
33	0	Amp	NR	NR	NR	NP

Amp: gene amplification; ap, atypical pattern (F, fused signals; G, green signals); CNG, copy number gain; NP, not performed; NR, not rearranged; wt, wild type.

### Comparison between the different methods

All 25 pan‐TRK IHC positive cases showed no fusions by FISH, RT‐PCR, and NGS testing. All six cases carrying *NTRK1* gene amplification and all cases with copy number gain of *NTRK1*, *NTRK2*, or *NTRK3* were pan‐TRK IHC negative. All cases showing *NTRK1* rearrangement in <15% of tumor cells analyzed were negative by RT‐PCR and NGS. In particular, all three cases harboring the *NTRK1* rearrangement in approximately 7% of the scattered neoplastic cells by FISH showed 3–10 on‐target reads by NGS, which is considered negligible for possible gene rearrangement. All cases that showed *NTRK1* split‐apart signals less than two signals in diameter in approximately 5–15% of tumor cells were negative by RT‐PCR and NGS. All cases that showed an atypical *NTRK1* FISH pattern were negative by RT‐PCR and NGS. Complete agreement was found between RT‐PCR and NGS results (Table 2).

## Discussion

*NTRK* gene fusions have been described in various tumor types with variable frequency. Tumors harboring *NTRK* fusions are sensitive to treatment with specific inhibitors regardless of histological features. To date, NTRK target inhibitors have been approved by the Food and Drug Administration (FDA), USA, for the treatment of TRK fusion‐positive cancers in a tumor agnostic way. Previous studies have reported that the incidence of *NTRK* gene fusions in breast carcinoma, not otherwise specified, is very low, ranging from 0 to 0.08% [17, 18, 19], whereas secretory breast carcinoma is characterized by *NTRK3*‐*ETV6* fusion [20]. Moreover, clinical trials have demonstrated that secretory breast carcinoma patients carrying *NTRK* fusions have a high response to TRK inhibitor therapy [21]. To the best of our knowledge, only two reports have evaluated *NTRK* fusions in TNBC and no clinical trial results are available to date [14, 17]. The ESMO guidelines recommend NTRK IHC as a screening method to select cases for additional molecular testing to evaluate the fusions [7]. Pan‐TRK IHC represents a useful screening test since it is feasible in most pathology laboratories at moderate cost allowing the inclusion of all potential positive cases in triage for molecular testing. Several studies have analyzed the sensitivity and specificity of NTRK IHC, with very different results. In the literature, some studies have reported a sensitivity of pan‐TRK IHC of approximately of 100% [22, 23]. Other data showed lower sensitivity to detect *NTRK* fusions by IHC, particularly in the studies reported by Solomon *et al* (88%) [9], Hondelink *et al* (82%) [24], Koopman *et al* (79%) [12], and Gatalica *et al* (75%) [13]. A false‐negative IHC result was observed in particular for cases carrying *NTRK3* rearrangements, regardless of the tumor type [9, 12, 13, 24]. In our TNBC series, pan‐TRK IHC was not sensitive, since all 25 pan‐TRK positive cases were wild type by molecular testing. Similarly, different percentages of specificity have been reported frequently according to the type of tumor. Solomon *et al* showed a specificity of 100% for carcinomas of the colon, lung, thyroid, pancreas, and biliary tract, with a lower specificity in breast and salivary gland carcinomas, 82 and 52%, respectively [9]. Since pan‐TRK IHC negative patients lose the possibility of further molecular investigation in clinical practice, good technical and interpretative practices for pan‐TRK IHC are recommended to avoid false negative results [25]. Our results confirm that pan‐TRK IHC has high specificity and negative predictive value for the detection of *NTRK* gene fusions. In our series, 14 of 25 pan‐TRK positive cases (56%) displayed only weak immunoreaction and were wild type by molecular analysis, suggesting that the cases showing weak pan‐TRK expression could be disregarded, significantly reducing the need for, and costs of, further molecular tests.

Molecular testing remains the gold standard for *NTRK* fusion detection. However, several critical pitfalls must be considered in the choice and interpretation of the available tests. Historically, FISH has been used to identify gene fusion detection in clinical practice, as in the identification of *ALK*, *ROS1*, and *RET* rearrangements in lung cancer. Regarding *NTRK* FISH, no guidelines for interpretation have yet been provided, thus comparison studies with NGS and RT‐PCR could clarify the evaluation criteria. Several interpretive doubts are currently unresolved in the interpretation of *NTRK* FISH, i.e. the cutoff for defining a rearranged case, the atypical patterns, the distance of separation of the split‐apart signals [7, 17, 26, 27]. In our study, some cases with dubious FISH have been solved by comparison with NGS and RT‐PCR; in particular, all cases showing separation of the split‐apart signals by less than two signal diameters did not show fusions by the orthogonal assays suggesting the strict use of at least two signal diameters to define a true rearrangement. Regarding the FISH threshold, the two cases with pan‐TRK IHC positive (intensity score 1+) and *NTRK1* FISH showing about 7% of tumor cells rearranged were negative by NGS. Our and other previous findings related to *NTRK* FISH reveal the need for consensus criteria in the evaluation of this test in order to avoid false results. In our series, we identified four cases with an isolated 5′ signal pattern by *NTRK1* FISH. Furthermore, two of these four cases showed pan‐TRK IHC positive staining, while no fusion was documented by NGS. Similarly to *ALK* FISH interpretation, the loss of 3′ signal (isolated 5′ signal) is routinely considered negative in the interpretation of *NTRK* FISH. Although isolated 5′ signal *ALK* FISH is still currently considered negative, two patients carrying this atypical pattern have tested positive with NGS and showed complete response to crizotinib, a specific ALK inhibitor [28]. In our series, the cases with isolated 5′ signal *NTRK1* were negative by both RT‐PCR and NGS; however, the lesson learnt from *ALK* rearrangement suggests that the cases harboring atypical *NTRK* patterns in FISH should be better characterized through other orthogonal assays. In this context, the NGS approach provides high‐throughput data and the analysis of multiple genetic loci simultaneously. However, some samples are not suitable for this test related to the quantity and the quality of the RNA [29].

Beyond *NTRK* fusions, our findings show an increase in the copy number of *NTRK* genes in our TNBC series. Copy number gain was present for *NTRK1* and *NTRK2* with a frequency of 1.2% and for *NTRK3* in 6% of cases analyzed. Moreover, *NTRK1* gene amplification was present in 6 of 83 cases (7.2%) but none of these cases showed pan‐TRK positive staining. The amplification of ‘driver’ genes represents a frequent mechanism related to tumorigenesis through the deregulation of both normal growth and survival pathways in cancer [30]. *NTRK* gene amplification could play a role in cancer, regardless of the presence of fusions. *NTRK* gene amplification was previously described in other solid tumors such melanoma, uterine leiomyosarcoma, squamous cell lung cancer and gastric adenocarcinoma [10]. Lee *et al* have analyzed a total of 1,250 tumor specimens and detected *NTRK* amplification in 28 cases of various types of cancer. Among these cases, only four were positive for pan‐TRK IHC, including one melanoma, one sarcoma, one lung cancer, and one gastric cancer [10]. These data demonstrated that *NTRK* gene amplification does not necessarily result in protein overexpression, as found in all our TNBCs. Similarly to our findings, Lee *et al* showed that the amplification mainly affects *NTRK1* rather than *NTRK2* and *NTRK3*. However, they used a different threshold for defining gene amplification; specifically, we used a cutoff of mean copy number gene/cell ≥6.0 copies, while Lee *et al* used a mean copy number of gene/cell of ≥4.0 copies. Beyond the mean number of gene copies per cell it is mandatory for the correct analysis of gene amplification to evaluate the ratio between the gene copies and the relative centromere to exclude polysomy. From this point of view, we have demonstrated disomy of chromosome 1 in our *NTRK1* amplified cases. Until now, few data have been reported regarding the sensitivity to NTRK‐specific inhibitors of patients harboring *NTRK* amplification regardless of fusions [31]. Currently, the phase II clinical trial NCT04879121 is recruiting patients with *NTRK* gene amplification solid tumors that are locally advanced or metastatic to evaluate the effect of larotrectinib in terms of overall response rate [32]. Further data are needed about *NTRK* amplification to understand its possible clinical significance, it is being understood that to date only tumors carrying *NTRK* fusions have been shown to benefit from treatment with specific inhibitors.

In conclusion, different from other tumors, pan‐TRK IHC showed a high false‐positive rate in TNBC for the detection of *NTRK* fusions, as determined by molecular testing. Our findings show that the *NTRK* genes are not involved in fusions in TNBC, but both copy number gain and amplification of *NTRK* genes are frequent events with an unknown potential predictive role. The role of *NTRK* gene amplification as an oncogenic driver will be clarified by the results of future clinical trial [32]. Moreover, our data provide useful guidelines for *NTRK* FISH interpretation suggesting the use of a stringent cut‐off for positivity of at least >15% of the cells analyzed and a mandatory separation distance of the split‐apart signals of two signal diameters to define a real gene fusion. Finally, the finding of an isolated 5′ signal remains an open issue, thus great attention must be paid in clinical practice to these cases.

## Author contributions statement

FZM, MA and RF conceived the study. SB, RG, FM, GA, GC, GV, AS, LI, FF and MO collected samples. MA, MM, RG, GV and RF evaluated the pathology. SB and FZM carried out experiments. MA, MM, RG, GV and RF performed analyses. FZM, MA and RF interpreted data. FZM and RF drafted the first version of the manuscript. FZM, MA and RF reviewed and edited the manuscript. All authors have read and agreed to the published version of the manuscript.

## Informed consent statement

The study was conducted in accordance with the Declaration of Helsinki and approved by the Institutional Review Board of University of Campania ‘L. Vanvitelli’‐AORN ‘Ospedale dei Colli’ Naples, Italy with approval number 21 of 17 January 2020.

## Supporting information


**Figure S1.** Representative results from a secretory breast carcinoma used as a positive control
**Table S1.** Panel of gene fusions detected but not distinguishable by RT‐PCR

## Data Availability

The data that support the findings of this study are available from the corresponding author upon reasonable request.
