# Supplementary material for: NTRK gene aberrations in triple‐negative breast cancer: detection challenges using IHC, FISH, RT‐PCR, and NGS
Source: J Pathol Clin Res. 2023 May 4;9(5):367–77. doi: 10.1002/cjp2.324 (PMC10397374; doi:10.1002/cjp2.324)
Supplement: Supplementary file 1 — Figure S1. Representative results from a secretory breast carcinoma used as a positive control Table S1. Panel of gene fusions detected but not distinguishable by RT‐PCR [file CJP2-9-367-s001.pdf]

NTRK gene aberrations in triple-negative breast cancer: detection challenges using IHC, FISH, RT-PCR, and NGS

F Zito Marino *et al*., *J Pathol Clin Res,* https://doi.org/10.1002/cjp2.324

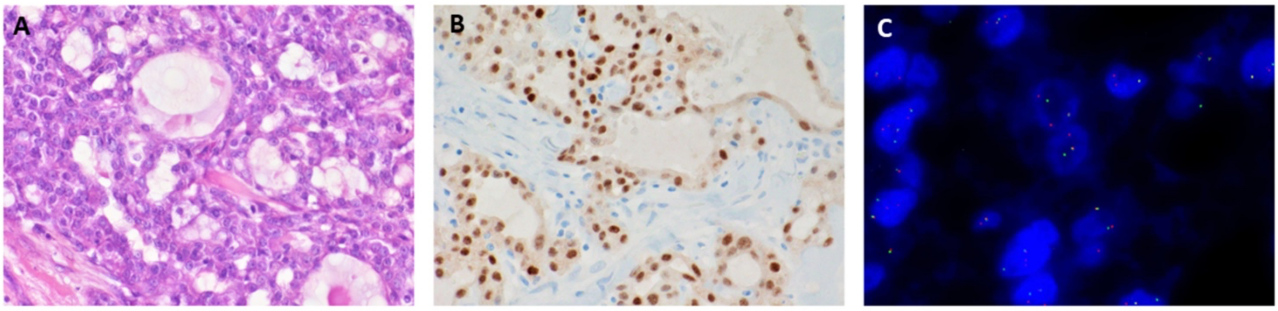

Figure S1. Representative results from a secretory breast carcinoma used as a positive control: (A) hematoxylin and eosin staining (original magnification ×40); (B) Pan-TRK IHC showing strong nuclear immunoreactivity in neoplastic cells (original magnification ×40); (C) *NTRK3* FISH showing the typical pattern of rearrangement: one fusion signal and split 3’ and 5’ signals, with a separation distance of at least two signal diameters between the green and orange signals (original magnification ×100).

Table S1. Panel of gene fusions detected but not distinguishable by RT-PCR.

Target	Exon	Gene fusion
NTRK1	exon 9–10	TFG-NTRK1 T5:N9
		NFASC-NTRK1 N20:N10
		NFASC-NTRK1 N21:N10
	exon 10	SQSTM1-NTRK1 S4:N10
		TFG-NTRK1 T5:N10
		TPM3-NTRK1 T7:N10
		TPR-NTRK1 T21:N10
		TRIM63-NTRK1 T8:N10
	exon 11–12 del	BCAN-NTRK1 B13:N11
		LMNA-NTRK1 L11 del178:N11
		LMNA-NTRK1 L2:N11
		PPL-NTRK1 P22 del2181:N11
		TPR-NTRK1 T6 del122:N12 del99
	exon 12	MPRIP-NTRK1 M21:N12
		MPRIP-NTRK1 M18:N12
		MPRIP-NTRK1 M14:N12
		SCYL3-NTRK1 S11:N12
		TPM3-NTRK1 T7:N1
		TPR-NTRK1 T6:N12
NTRK2	exon 12–15	AFAP1-NTRK2 A13:N12
		VCL-NTRK2 V16:N12
		TLE4-NTRK2 T7:N15
		TRIM24-NTRK2 T12:N15
	exon 16–17	AGBL4-NTRK2 A6:N16
		QKI-NTRK2 Q6:N16
		SQSTM1-NTRK2 S4:N16
		STRN-NTRK2 S3:N16
		SQSTM1-NTRK2 S4:N17
NTRK3	exon 14	ETV6-NTRK3 E4:N14
		ETV6-NTRK3 E5:N14
	exon 15	ETV6-NTRK3 E4:N15
		ETV6-NTRK3 E5:N15
